# Size dependence of structural parameters in fcc and hcp Ru nanoparticles, revealed by Rietveld refinement analysis of high-energy X-ray diffraction data

**DOI:** 10.1038/srep31400

**Published:** 2016-08-10

**Authors:** Chulho Song, Osami Sakata, Loku Singgappulige Rosantha Kumara, Shinji Kohara, Anli Yang, Kohei Kusada, Hirokazu Kobayashi, Hiroshi Kitagawa

**Affiliations:** 1 Synchrotron X-ray Station at SPring-8, Research Network and Facility Services Division, National Institute for Materials Science, 1-1-1 Kouto, Sayo, Hyogo 679-5148, Japan; 2 Synchrotron X-ray Group, Research Center for Advanced Measurement and Characterization, National Institute for Materials Science, 1-1-1 Kouto, Sayo, Hyogo 679-5148, Japan; 3 Department of Innovative and Engineered Materials, Tokyo Institute of Technology, 4259-J3-16, Nagatsuta, Midori, Yokohama 226-8502, Japan; 4 Division of Chemistry, Graduate School of Science, Kyoto University, Kitashirakawa Oiwake-cho, Sakyo-ku, Kyoto 606-8502, Japan; 5 Institute for Integrated Cell-Material Sciences, Kyoto University, Yoshida, Sakyo-ku, Kyoto 606-8501, Japan; 6 INAMORI Frontier Research Center, Kyushu University, 744 Motooka, Nishi-ku, Fukuoka 819-3095, Japan

## Abstract

To reveal the origin of the CO oxidation activity of Ruthenium nanoparticles (Ru NPs), we structurally characterized Ru NPs through Rietveld refinement analysis of high-energy X-ray diffraction data. For hexagonal close-packed (hcp) Ru NPs, the CO oxidation activity decreased with decreasing domain surface area. However, for face-centered cubic (fcc) Ru NPs, the CO oxidation activity became stronger with decreasing domain surface area. In comparing fcc Ru NPs with hcp Ru NPs, we found that the hcp Ru NPs of approximately 2 nm, which had a smaller domain surface area and smaller atomic displacement, showed a higher catalytic activity than that of fcc Ru NPs of the same size. In contrast, fcc Ru NPs larger than 3.5 nm, which had a larger domain surface area, lattice distortion, and larger atomic displacement, exhibited higher catalytic activity than that of hcp Ru NPs of the same size. In addition, the fcc Ru NPs had larger atomic displacements than hcp Ru NPs for diameters ranging from 2.2 to 5.4 nm. Enhancement of the CO oxidation activity in fcc Ru NPs may be caused by an increase in imperfections due to lattice distortions of close-packed planes and static atomic displacements.

Ruthenium (Ru) has recently attracted much attention as a catalyst for the oxidation of CO because of its high catalytic activity^1^,^2^,^3^,^4^,^5^. This phenomenon was reported in 2013 for face-centered-cubic (fcc) type Ru nanoparticles (NPs) obtained by chemical reduction^6^. However, the dependence of properties of the fcc Ru on lattice parameters was analyzed by ab initio calculations^7^, although naturally occurring bulk Ru forms only a hexagonal close-packed (hcp) structure. The structure and crystal size are controlled by adjusting the composition of mixtures of the Ru precursor and reducing agent^6^. Moreover, the catalytic activity of Ru NPs, supported on *γ*-Al_2_O_3_ for CO oxidation, is dependent on the fcc and hcp structure and size^6^. For NPs larger than 3 nm, the newly obtained fcc Ru NPs are more reactive than conventional hcp Ru NPs^6^. Counterintuitively, the catalytic activity of the fcc Ru NPs increases with increasing particle size, despite the decrease in surface area^6^.

According to a review of the structures of Ru NPs smaller than 5 nm, two approaches have been pursued. Phase identification and crystalline domain size have been determined from the peak widths obtained by X-ray diffraction (XRD), by using laboratory-based X-ray instruments^6^,^8^,^9^. Another study^10^ has revealed atomic-scale three-dimensional configurations in hcp Ru NPs, by using a high-energy X-ray source (90.48 keV), reverse Monte Carlo simulations, and atomic pair distribution function analyses. However, there has been no report on the relationship between the mean structure and CO oxidation activity of fcc Ru NPs.

In this study, we present the structures of both fcc- and hcp-type Ru NPs, determined through Rietveld analysis of high-energy X-ray diffraction data. To help reveal the origin of the CO oxidation activity of Ru NPs, we used the results of the Rietveld analysis to evaluate the dependence of lattice distortion, domain surface area, and static atomic displacement on particle size.

## Methods

### Sample preparation

Eight samples were investigated (Table 1). The fcc and hcp Ru NPs were fabricated by chemical reduction methods using Ru(acac)_3_ and RuCl_3_ · nH_2_O, respectively, as the metal precursors, and poly(N-vinyl-2-pyrrolidone) (PVP) as the stabilizing agent; the detailed procedure has been described in ref. 6. Size control was achieved by adjusting the concentrations of the reagents (triethylene glycol (TEG) or ethylene glycol (EG)) and the PVP stabilizer used in the synthesis. However, the shape of the Ru NPs was not controlled. As shown in the transmission electron microscopy (TEM) image (see Supplementary Information Fig. S1(a)), fcc Ru NPs can have several shapes, including decahedra, icosahedra, and truncated pyramids. The size of the prepared samples was determined from TEM images. The details for the determination of size and temperature for 50% conversion of CO to CO_2_ (T_50_) are also described in ref. 6.

### High-energy X-ray diffraction experiments

High-energy XRD experiments were performed at beamline BL04B2 at SPring-8 in Japan, using X-rays of energy 61.46 keV. The samples were as-prepared Ru NPs instead of those supported on *γ*-Al_2_O_3_ and were not subjected to the reactive gaseous environment. Information about the oxidation states of catalysts under the given reaction conditions was not obtained. It is therefore difficult to assess the direct relation between their structural characterization and CO oxidation activity. However, we focused on the structural analysis of Ru nanoparticles before CO oxidation. The measurements were performed in symmetric transmission geometry, and the diffraction patterns were collected at intervals of 0.1° and exposure times of 10 s per point for the small-angle regime of 2*θ* = 1~10°; intervals of 0.15° and exposure times of 20 s for the middle-angle regime of 2*θ* = 9~20°;and intervals of 0.2° and exposure times of 40 s for the large-angle regime of 2*θ* = 19~48.2°.

## Results and Discussion

**Figure 1:**
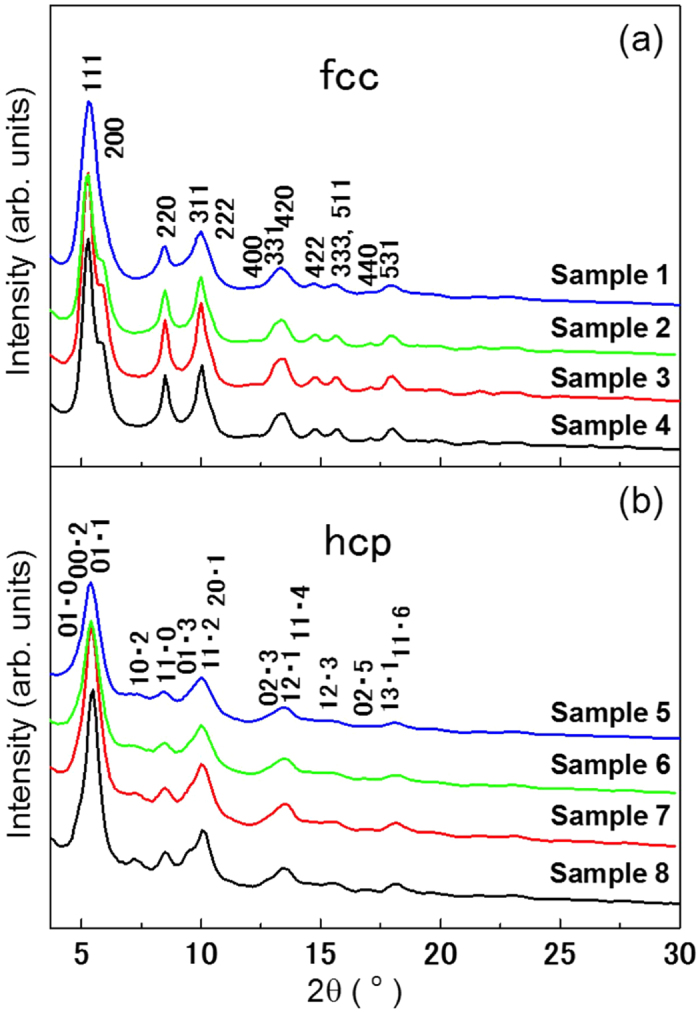
XRD patterns for (**a**) fcc and (**b**) hcp Ru NPs at room temperature. The incident X-ray energy used was 61.46 keV. Five Bragg peaks (111, 200, 220, 311, and 222) for the fcc Ru NPs and six Bragg peaks (01 · 0, 00 · 2, 01 · 1, 10 · 2, 11 · 0, and 01 · 3) for the hcp Ru NPs were used in determining the average crystalline domain size, as shown in Fig. 2.

Figure 1 shows the XRD patterns for all of the as-prepared Ru NPs. For fcc Ru NPs, the full width at half maximum (FWHM) of the diffraction peaks became sharper from Sample 1 to Sample 2. In particular, the single broad peak in Sample 1 was clearly divided into a 111 main peak and 200 shoulder peak in Sample 2. The FWHM of the diffraction peaks did not undergo a considerable change from Sample 2 to Sample 4. This result suggested that there was a considerable change in the crystalline domain size from Sample 1 to Sample 2, and Samples 2, 3, and 4 had almost the same size. For hcp Ru NPs, we observed that as the FWHM of the diffraction peaks broadened, the mean particle size, obtained from TEM observations, decreased.

All XRD patterns for the Ru NPs were analyzed using the Rietveld refinement method^11^ for a pseudo-Voigt function. The results of the Rietveld refinement analysis are presented in Fig. S2 of the Supplementary Information. The plot shows the refined XRD patterns, residuals, positions of the Bragg peaks, and agreement factors of *R*_*wp*_ and *R*_*B*_. In this refinement, the Ru fractional position and occupancy were fixed. Other parameters, such as lattice constants, *B* factor, scale factor, and peak-shape function parameters, were taken as free parameters. The *B* factor is defined as the mean squared displacement and is related to atomic thermal vibrations. The space groups associated with hcp and fcc Ru NPs are P6_3_/mmc and 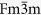, respectively. The phase identification of all the Ru NPs, determined from Rietveld analysis, was in agreement with that obtained from high-resolution TEM (HRTEM) images given in ref. 6. In addition to the phase identification, the evaluated average domain size, lattice parameters, unit-cell volume, number of unit cells, number of domains, *B* factor, lattice distortion, and domain surface area are listed in Tables 2 and 3. Notably, the lattice parameter of fcc Ru NPs was larger than the theoretical value of fcc Ru bulk (*a* = 3.83 Å)^7^ and tended to increase with decreasing particle size. In contrast, the lattice parameters of hcp Ru NPs were smaller than those for hcp bulk Ru (*a* = 2.7058(2) Å, *c* = 4.2816(7) Å)^12^, and tended to contract with decreasing particle size, except for sample 6. This size effect on the lattice parameters of metallic NPs was in agreement with results from previous studies^13^,^14^,^15^,^16^,^17^,^18^,^19^.

**Figure 2:**
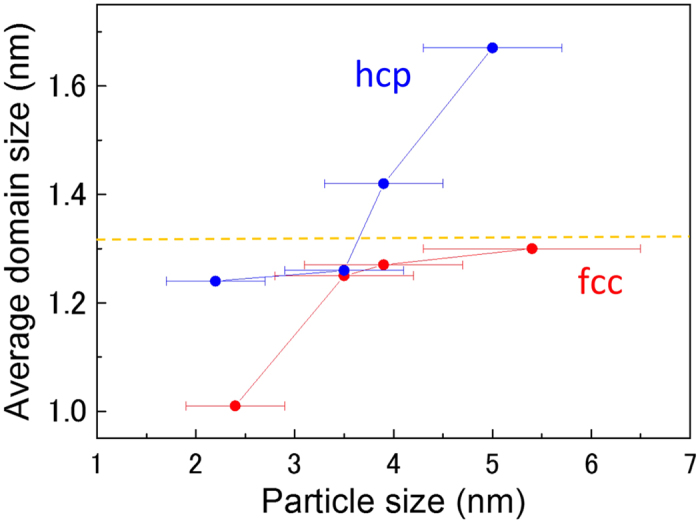
Average crystalline domain size as a function of particle size. The dashed line shows the limit of growth of crystalline domains for fcc Ru NPs.

Figure 2 shows the relationship between the average crystalline domain size and particle size. The domain size was calculated from the Scherrer equation, *D* = *Kλ*/*β* cos *θ*, where *D* is the average crystalline domain size, *K* (=0.9 if nanoparticles are assumed to be spherical) is the shape factor, *λ* (= 0.202 Å) is the X-ray wavelength, *β* is the line-broadening of an observed peak, expressed as FWHM in radians, and *θ* is the Bragg angle. Here, the average crystalline domain sizes were obtained using five Bragg peaks (111, 200, 220, 311, and 222) for the fcc Ru NPs and six Bragg peaks (01 · 0, 00 · 2, 01 · 1, 10 · 2, 11 · 0, and 01 · 3) for the hcp Ru NPs. The accuracy of the Scherrer equation is limited by the uncertainties in *β*. The errors of *β*, determined from the results of the Rietveld analysis, were approximately 10%.

For hcp Ru NPs, the average domain size increased linearly with increasing particle size. In contrast, for fcc Ru NPs, the average domain size saturated at approximately 1.3 nm despite the increase in particle size. These results were consistent with those expected from Fig. 1.

**Figure 3:**
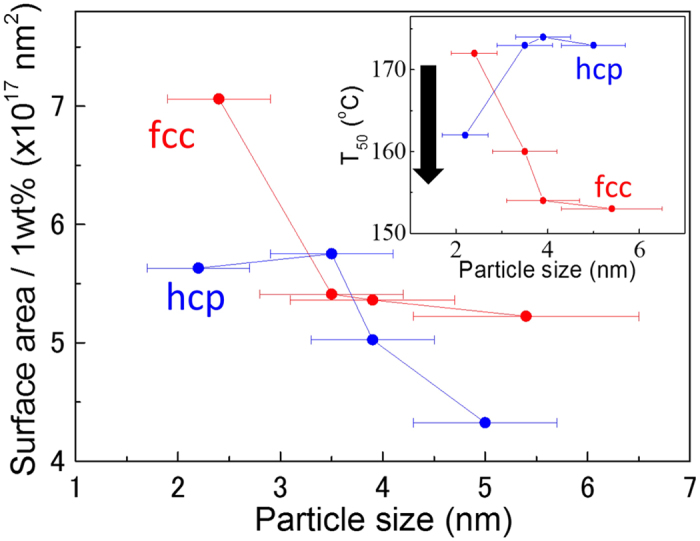
Dependence of surface area of a crystalline domain (obtained from the Rietveld analysis) on particle size. Reprinted with permission from Fig. 4 page 5495 in vol. 135 Copyright 2013 American Chemical Society. The inset shows the temperature dependence for 50%-conversion of CO to CO_2_ (T_50_) as a function of particle size, as reported in ref. 6. The direction of the arrow indicates higher CO oxidation capability.

The inset in Fig. 3 shows that for the hcp Ru NPs, T_50_ increased significantly from 2.2 nm to 3.5 nm and was stable above 3.5 nm. In contrast, for the fcc Ru NPs, T_50_ decreased with particle size. This result indicates that fcc Ru NPs are more reactive than the conventional hcp Ru NPs larger than 3.5 nm in particle size. Catalytic characterizations (T_50_) of the synthesized Ru NPs, supported on *γ*-Al_2_O_3_ were carried out by using a tubular quartz reactor. The details of catalyst preparation and catalytic characterization are described in sections 1 and 2 of the Supplementary Information. To reveal the origin of the higher activity of the fcc Ru NPs, we plotted the relationships between the surface area of crystalline domain/lattice distortion/*B* factor and particle size.

Figure 3 shows the relationship between the surface area of the crystalline domain (obtained from Rietveld analysis) and particle size (obtained from TEM results). The surface area was calculated with the following equation: *A*_*surface*_ = *N* × 4*π*(*D*/2)^2^. Here, *A*_*surface*_ denotes the surface area of the crystalline domain, *N* is the number of domains per 1.5 mg of Ru NPs (equivalent to 1 *wt*% of *γ*-Al_2_O_3_), and *D* is the average crystalline domain size. *N* is defined by the following equation: *N* = (Number of Ru atoms per 1.5 mg) ÷ (Number of Ru atoms per domain). The details for the calculation of *N* are described in section 3 of the Supplementary Information. In general, CO-catalytic activity was expected to be proportional to the surface area of the NPs. For hcp Ru NPs, the catalytic activity diminished with decreasing surface area. In contrast, for fcc Ru NPs, the catalytic activity was enhanced with deceasing surface area. When we compared fcc Ru NPs with hcp Ru NPs of the same particle size, we found that the hcp Ru NPs with a smaller crystalline domain area showed higher catalytic activity than did fcc Ru NPs, for small particle sizes of approximately 2 nm. The higher activity of the 2.2 nm-diameter hcp Ru NPs may have originated from the contribution of particle surfaces. In contrast, for NPs larger than 3.5 nm in particle size, the fcc Ru NPs showed larger surface areas and higher catalytic activities than the hcp Ru NPs.

**Figure 4:**
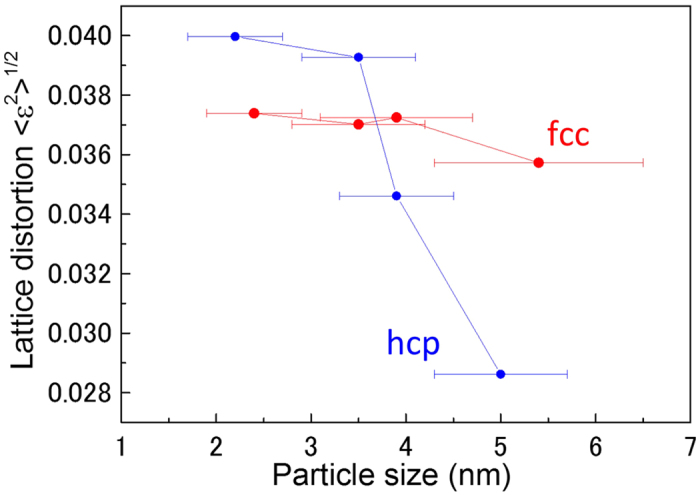
Dependence of lattice distortion on particle size. In contrast with hcp Ru NPs, the lattice distortion of fcc Ru NPs was nearly independent of particle size.

Figure 4 shows the relationship between the lattice distortion ratio and particle size. The lattice distortion was determined by using the (111) plane of the fcc Ru NPs and the (002) plane of the hcp Ru NPs. The mechanism of CO oxidation with hcp Ru has been reported to begin with the oxidation of Ru (001), thus forming several RuO_2_ (1110) layers, after which CO oxidation occurs on RuO_2_ (110)^20^,^21^,^22^. The fcc Ru (111) planes are also close-packed in a manner similar to the hcp Ru (001) planes. The crystal lattice distortion 〈*ε*^2^〉^1/2^ of Ru NPs at various domain sizes was calculated from the following equation^23^,^24^:


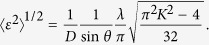


The distortion is the root mean square (rms) microstrains averaged along the [h k l] direction^25^. Clearly, 〈*ε*^2^〉^1/2^ depends only on *D* for fixed Bragg angles and 〈*ε*^2^〉^1/2^ decreases as *D* increases.

The data in Fig. 4 suggest a decrease in the lattice distortion with increasing particle size for hcp Ru NPs. However, the lattice distortion was nearly independent of particle size for fcc Ru NPs. When we compared fcc Ru NPs with hcp Ru NPs of the same particle size, we found that the lattice distortions in the hcp Ru NPs were larger than those in the fcc Ru NPs for small-sized particles below 3.5 nm. In contrast, the lattice distortions of the fcc Ru NPs were considerably larger than those of the hcp Ru NPs of large sizes (above 3.5 nm). The higher catalytic activity of the fcc Ru NPs can be attributed to this result, along with the result in Fig. 3.

**Figure 5:**
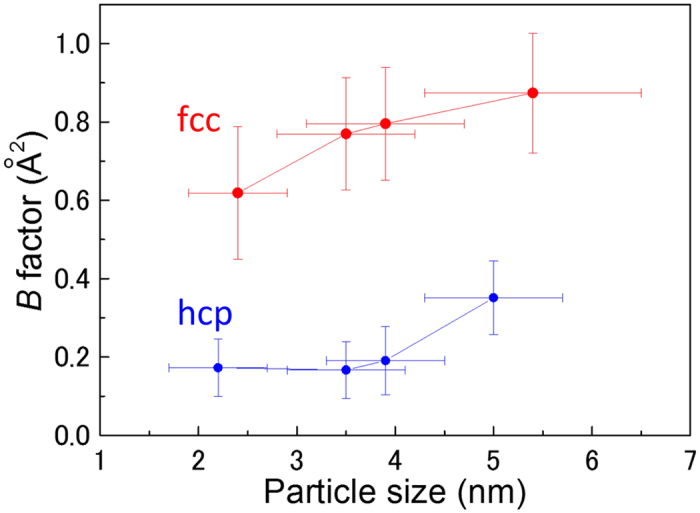
*B* factor as a function of particle size. Notably, the *B* factor of hcp Ru NPs is considerably smaller than that of the fcc Ru NPs.

Figure 5 shows the particle size-dependence of the *B* factor. The error bars for the *B* factor were determined from the results of the Reitveld analysis. The *B* factor is defined as^26^: 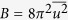, where 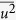 is the mean squared displacement, and can be regarded as an indication of the relative thermal vibrational motions, which is related to static atomic displacement. Atoms with a small *B* factor belong to a part of the structure that is well ordered. In contrast, atoms with a large *B* factor generally belong to a part of the structure that is very flexible or is more reactive to an ambient environment. The *B* factor of the fcc and hcp Ru NPs increased slightly with particle size. When comparing fcc Ru NPs and hcp Ru NPs of a similar particle size, we found that the fcc Ru NPs with a large *B* factor showed high catalytic activity for large particles of size above 3 nm. In contrast, hcp Ru NPs with a small *B* factor showed high catalytic activity for small particles with a size of approximately 2 nm. This result was far from being obvious from physical considerations, similarly to the result of the domain surface area for small particles of size approximately 2 nm, as shown in Fig. 3. However, as shown in Fig. 4, hcp Ru NPs with a larger lattice distortion showed high catalytic activity for small particles of size approximately 2 nm. Consequently, for these small Ru NPs, lattice distortion might have a greater effect on the catalytic activity, rather than the domain surface area and the *B* factor. For hcp Ru NPs, the catalytic activity appeared to diminish as the *B* factor increased. However, the *B* factor of the hcp Ru NPs was considerably smaller than that of the fcc Ru NPs. For fcc Ru NPs, the catalytic activity increased as the *B* factor increased.

## Conclusion

We determined the crystallographic information such as average crystalline domain size, domain surface area, lattice distortion, and B factor to reveal the origin of the CO oxidation activity of Ru NPs. The information was obtained from Rietveld refinement analysis of high-energy X-ray diffraction data. *B* factor and lattice distortion were more correlated to the CO oxidation activity. For small particles with sizes below 3.5 nm, lattice distortions of the close-packed plane in the hcp Ru NPs were greater than those in the fcc Ru NPs. In contrast, for large NPs with sizes above 3.5 nm, lattice distortions of the close-packed plane and *B* factor values, which is related to static atomic displacement, in the fcc Ru NPs were greater than those for the hcp Ru NPs. Although these results were obtained through a non-surface sensitive scattering technique, they may hold clues to understanding the higher CO oxidation activity of fcc Ru NPs compared with that of hcp Ru NPs, for sizes larger than 3 nm. Except for Ru NPs of approximately 2 nm, the large B factor affected the intensification of catalytic activity. In addition, for fcc Ru NPs, the *B* factor can have a larger influence than lattice distortions on the intensification of the catalytic activity with increasing particle size. Such structural information should contribute to improving the functionality of nano-sized catalysts.

## Additional Information

**How to cite this article**: Song, C. *et al*. Size dependence of structural parameters in fcc and hcp Ru nanoparticles, revealed by Rietveld refinement analysis of high-energy X-ray diffraction data. *Sci. Rep.* **6**, 31400; doi: 10.1038/srep31400 (2016).

## Supplementary Material

Supplementary Information

## Figures and Tables

**Table 1:** Reaction conditions for the synthesis of fcc and hcp Ru NPs and their catalytic activities T_50_ 6 (temperature for 50% conversion of CO to CO_2_).

Sample	Structure	Size (TEM)/nm	Metal precursor/mmol	Solvent/mL	PVP/mmol	T_50_ (°C)
1	fcc	2.4 ± 0.5	Ru(acac)_3_/2.1	TEG/500	10.0	172
2	fcc	3.5 ± 0.7	Ru(acac)_3_/2.1	TEG/200	10.0	160
3	fcc	3.9 ± 0.8	Ru(acac)_3_/2.1	TEG/100	5.0	154
4	fcc	5.4 ± 1.1	Ru(acac)_3_/2.1	TEG/25	1.0	153
5	hcp	2.2 ± 0.5	RuCl_3_ · nH_2_O/2.1	EG/500	10.0	162
6	hcp	3.5 ± 0.6	RuCl_3_ · nH_2_O/2.1	EG/200	10.0	173
7	hcp	3.9 ± 0.6	RuCl_3_ · nH_2_O/2.1	EG/100	5.0	174
8	hcp	5.0 ± 0.7	RuCl_3_ · nH_2_O/2.1	EG/25	1.0	173

Notably, the fcc Ru NPs were fabricated by chemical reduction methods using Ru(acac)_3_.

**Table 2:** Average crystalline domain size, lattice parameter, unit volume, number of unit cells, number of domains, *B* factor, lattice distortion, and domain surface area in hcp Ru NPs obtained using the Rietveld refinement method.

Sample	1	2	3	4
Crystalline domain size (nm)	1.01	1.25	1.27	1.30
*a* (Å)	3.9113 ± 0.0045	3.8743 ± 0.0029	3.8670 ± 0.0029	3.8706 ± 0.0032
Unit volume (Å^3^)	59.8357	58.1527	57.8273	57.9858
Number of unit cells	8.90	17.79	18.53	19.93
Number of domains (×10^16^)	22.03	11.02	10.58	9.84
*B* factor (Å^2^)	0.62 ± 0.17	0.77 ± 0.14	0.80 ± 0.14	0.87 ± 0.15
Lattice distortion at (111) plane	0.0374	0.0370	0.0373	0.0357
Domain surface area (×10^17^ nm^2^)	7.059	5.411	5.362	5.224

**Table 3:** Average crystalline domain size, lattice parameter, unit volume, number of unit cells, number of domains, *B* factor, lattice distortion, and domain surface area in fcc Ru NPs obtained using the Rietveld refinement method.

Sample	5	6	7	8
Crystalline domain size (nm)	1.24	1.26	1.42	1.67
*a* (Å)	2.6478 ± 0.0018	2.6814 ± 0.0019	2.6542 ± 0.0019	2.6657 ± 0.0016
*c* (Å)	4.1469 ± 0.0058	4.2175 ± 0.0060	4.1803 ± 0.0062	4.2260 ± 0.0052
Unit volume (Å^3^)	25.1773	26.2607	25.5041	26.0054
Number of unit cells	39.65	40.07	58.25	93.61
Number of domains (×10^16^)	11.66	11.53	7.93	4.94
*B* factor (Å^2^)	0.17 ± 0.07	0.17 ± 0.07	0.19 ± 0.09	0.35 ± 0.09
Lattice distortion at (002) plane	0.0400	0.0393	0.0346	0.0286
Domain surface area (×10^17^ nm^2^)	5.630	5.751	5.025	4.325
