# Supplementary material for: Size dependence of structural parameters in fcc and hcp Ru nanoparticles, revealed by Rietveld refinement analysis of high-energy X-ray diffraction data
Source: Sci Rep. 2016 Aug 10;6:31400. doi: 10.1038/srep31400 (PMC4979023; doi:10.1038/srep31400)
Supplement: Supplementary Information [file srep31400-s1.pdf]

Supplementary Information

Size dependence of structural parameters in fcc and hcp Ru nanoparticles, revealed by Rietveld refinement analysis of high-energy X-ray diffraction data

C. H. Song[1], O. Sakata[1, 2, 3,*], L. S. R. Kumara[1], S. Kohara[1, 2], A. Yang[1], K. Kusada[4], H. Kobayashi[4], and H. Kitagawa[4, 5, 6]

[1)] Synchrotron X-ray Station at SPring-8, National Institute for Materials Science, 1-1-1 Kouto, Sayo, Hyogo 679-5148, Japan

[2)] Synchrotron X-ray Group, Quantum Beam Unit, National Institute for Materials Science, 1-1-1 Kouto, Sayo, Hyogo 679-5148, Japan

[3)] Department of Innovative and Engineered Materials, Tokyo Institute of Technology, 4259-J3-16, Nagatsuta, Midori, Yokohama 226-8502, Japan

[4)] Division of Chemistry, Graduate School of Science, Kyoto University, Kitashirakawa Oiwake-cho, Sakyo-ku, Kyoto 606-8502, Japan

[5)] Institute for Integrated Cell-Material Sciences, Kyoto University, Yoshida, Sakyo-ku, Kyoto 606-8501, Japan

[6)] INAMORI Frontier Research Center, Kyushu University, 744 Motooka, Nishi-ku, Fukuoka 819-3095, Japan

[*]Author to whom correspondence should be addressed. E-mail: SAKATA.Osami@nims.go.jp

Section 1. Catalytic preparation

All the synthesized Ru nanoparticles were supported on γ-Al_2O_3 catalysts by wet impregnation. Each nanoparticle (equivalent to 1 *wt* % of γ-Al_2O_3) was ultrasonically dispersed in purified water. The γ-Al_2O_3 support has been pre-calcined at 800 °C for 5 h. The pre-calcined γ-Al_2O_3 was added into each nanoparticle solution, and then the suspended solutions were stirred for 12 h. After stirring, the suspended solutions were heated to 60 °C and dried under vacuum. The resulting powders were kept at 120 °C for 8 h for complete water removal. The obtained catalyst powders were pressed into pellets at 1.2 MPa for 5 min. The pellets were crushed and sieved to obtain grains with diameters between 180 and 250 μm.

Section 2. Catalytic characterization

For the investigation of catalytic activity in CO oxidation, each supported nanoparticle catalyst (150 mg) was loaded into a tubular quartz reactor (internal diameter, i.e. 7 mm) with quartz wool. A gas mixture of $CO/O_2/He$ ($He/CO/O_2$: 49/0.5/0.5 ml min^{-1}) was passed over the catalysts at ambient temperature, and the catalysts were then heated to 100 °C. After 15 min, effluent gas was collected, and the reaction products were analyzed by gas chromatography with a thermal conductivity detector (GC-8A, Shimadzu, Japan). The catalysts were heated in increments of 10 °C to the temperature at which CO was consumed completely, and the products were analyzed at each temperature. After the reaction, the reactor was purged with He at the reaction temperature, and the catalysts were then cooled to room temperature.

Section 3. Calculation of the number of domains (*N*) for evaluating the surface area of the crystalline domain ($A_{surface}$) for fcc and hcp Ru NPs

The number of domains for Ru NPs were calculated based on the same criteria of catalytic preparation and characterization in Section 1 and 2. Each Ru nanoparticle of 1.5mg (equivalent to 1 *wt* % of γ-Al_2O_3) was used in catalytic characterization. Molar mass of Ru is 101.07 *g/mol*. Therefore, the number of Ru atoms per 1.5 mg can be calculated by the following equation (eq. S1).

$$\text{Number of Ru atoms per 1.5mg} = \frac{1.5\times10^{-3}g}{101.07g} \times 6.02 \times 10^{23} \approx 8.94 \times 10^{18} \qquad \text{(eq. S1)}$$

The number of unitcell per domain can be calculated by the following equation (eq. S2).

$$\text{Number of unitcell per domain} = \frac{V_{domain}}{unit\ volume} = \frac{\frac{4}{3}\pi(\frac{D}{2})^3}{unit\ volume} \qquad \text{(eq. S2)}$$

where V_{domain} is the volume of crystalline domain, and *D* is the average crystalline domain size. Considering the number of Ru atoms included in unitcell for fcc and hcp Ru NPs, the number of Ru atoms per 1 domain can be simply calculated by the following equations (eq. S3 and eq. S4)

$$\text{Number of Ru atoms per domain (for fcc Ru)} = \text{Number of unitcell per domain} \times 4 \qquad \text{(eq. S3)}$$

$$\text{Number of Ru atoms per domain (for hcp Ru)} = \text{Number of unitcell per domain} \times 2 \qquad \text{(eq. S4)}$$

Finally, the number of domain (N) per 1.5 mg for fcc and hcp Ru NPs can be calculated from eq. S1, S3, and S4.

$$N = (\text{Number of Ru atoms per 1.5 mg}) \div (\text{Number of Ru atoms per domain}) \quad \text{(eq. S5)}$$

Section 4. TEM images for fcc and hcp Ru NPs

Figure S1 shows the TEM images for fcc Ru NPs (5.4 ± 1.1 nm) and hcp Ru NPs (5.0 ± 0.7 nm). Actually, we haven't yet controlled a shape of NPs. As you can be seen in Figure S1, Ru NPs can have several shapes, including decahedra, icosahedra, and truncated pyramids.

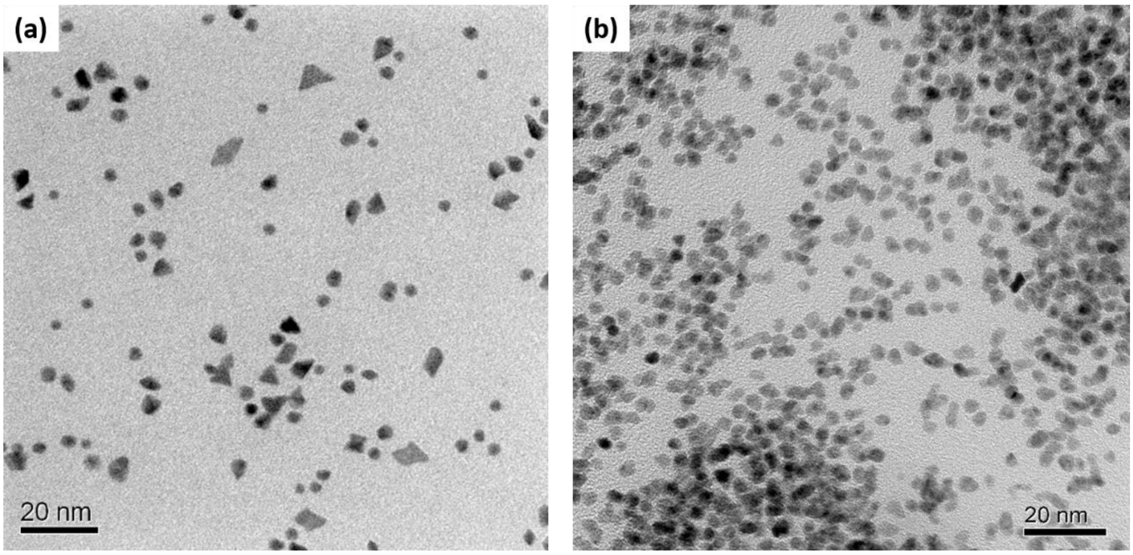

Figure S1. TEM images for (a) fcc Ru NPs: 5.4 ± 1.1 nm and (b) hcp Ru NPs: 5.0 ± 0.7 nm.

.

Section 5. Rietveld analysis for fcc and hcp Ru NPs

Figure S2 shows the results of the Rietveld refinement analysis for fcc and hcp Ru NPs at room temperature. As you can be seen in Figure S2, the experimental high-energy XRD patterns of fcc and hcp Ru NPs exhibit well-defined Bragg peaks that could be indexed in cubic and hexagonal unit cells, respectively.

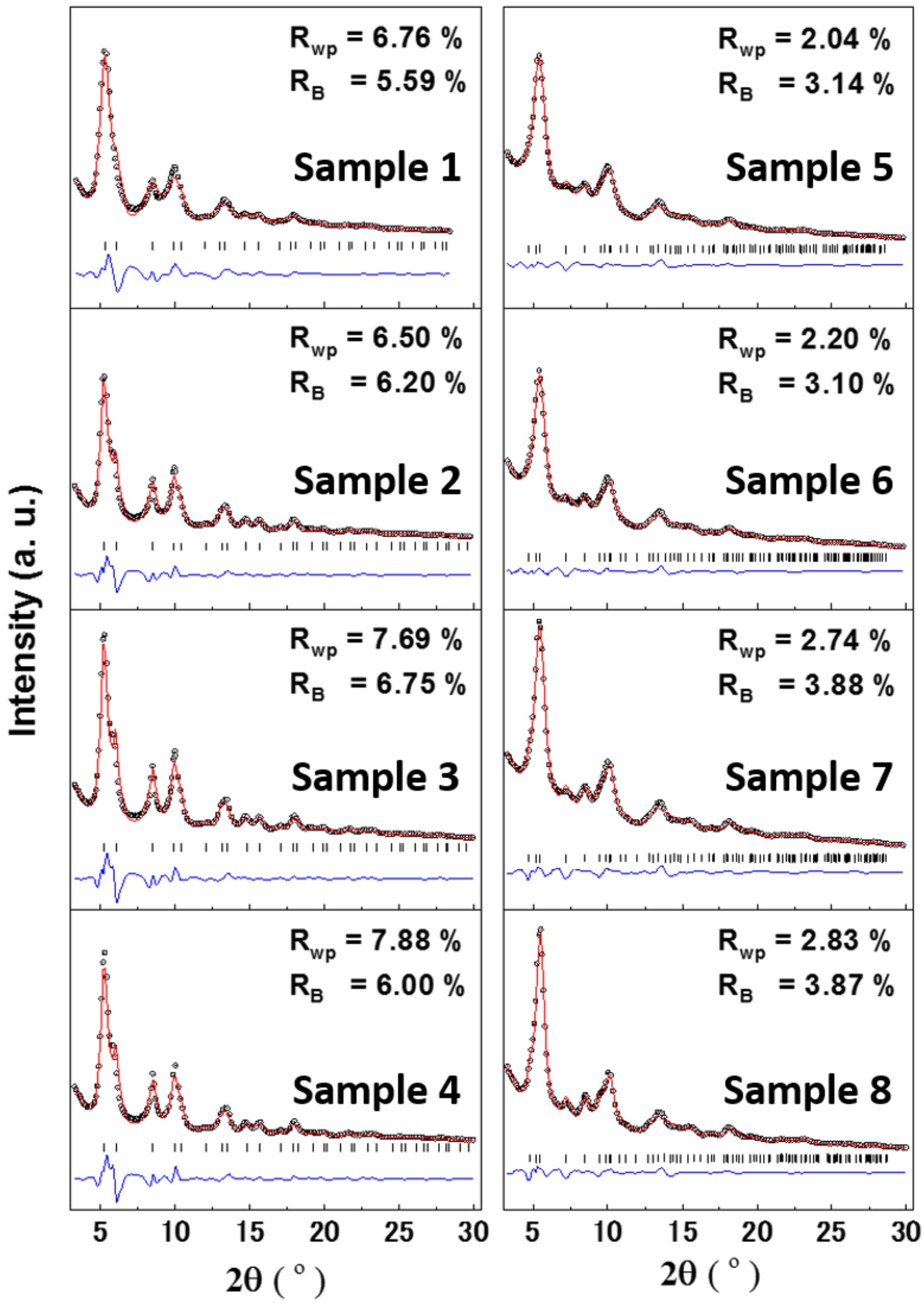

Figure S2. Refinement results for (a) fcc and (b) hcp Ru NPs at room temperature.
